# Supplementary material for: Defining quality by quantifying degradation in the mechanical recycling of polyethylene
Source: Nat Commun. 2024 Oct 9;15:8733. doi: 10.1038/s41467-024-52856-8 (PMC11464626; doi:10.1038/s41467-024-52856-8)
Supplement: Supplementary file 1 — Supplementary Information [file 41467_2024_52856_MOESM1_ESM.pdf]

Supplementary Information

Defining quality by quantifying degradation in the mechanical recycling of polyethylene

Arpan D. Patel[1,2], Zoé O. G. Schyns [1,2], Thomas W. Franklin [1,2], and Michael P. Shaver [1,2]*

[1] Department of Materials, School of Natural Sciences, University of Manchester, Manchester, M13 9BL, United Kingdom

[2] Sustainable Materials Innovation Hub, Henry Royce Institute, University of Manchester, Manchester, M13 9BL, United Kingdom

Number of Figures: 23

Number of Equations: 8

Contents

1)	Polyolefin Extrusions	2
2)	Crossover Modulus	3
3)	Gas-Mediated Simulated Recycling of polyolefins in gaseous environments.	4
4)	Flow rate variation	9
5)	Degradation limit determination for HDPE	11
6)	Rheological Dependence of Van Gurp-Palmen Plot	13
7)	Qualification of Vdeg in linear polymer melts	15
8)	Mixed gases apparatus	15
9)	Extrusion of polyolefins under gaseous environments	15
10)	Simulated extrusion of differing grades of HDPE	17
11)	Linear viscoelastic region determination and reproducibility within Van Gurp-Palmen plots	24
12)	Time-temperature superposition of Van Gurp-Palmen plots	25

1) Polyolefin Extrusions

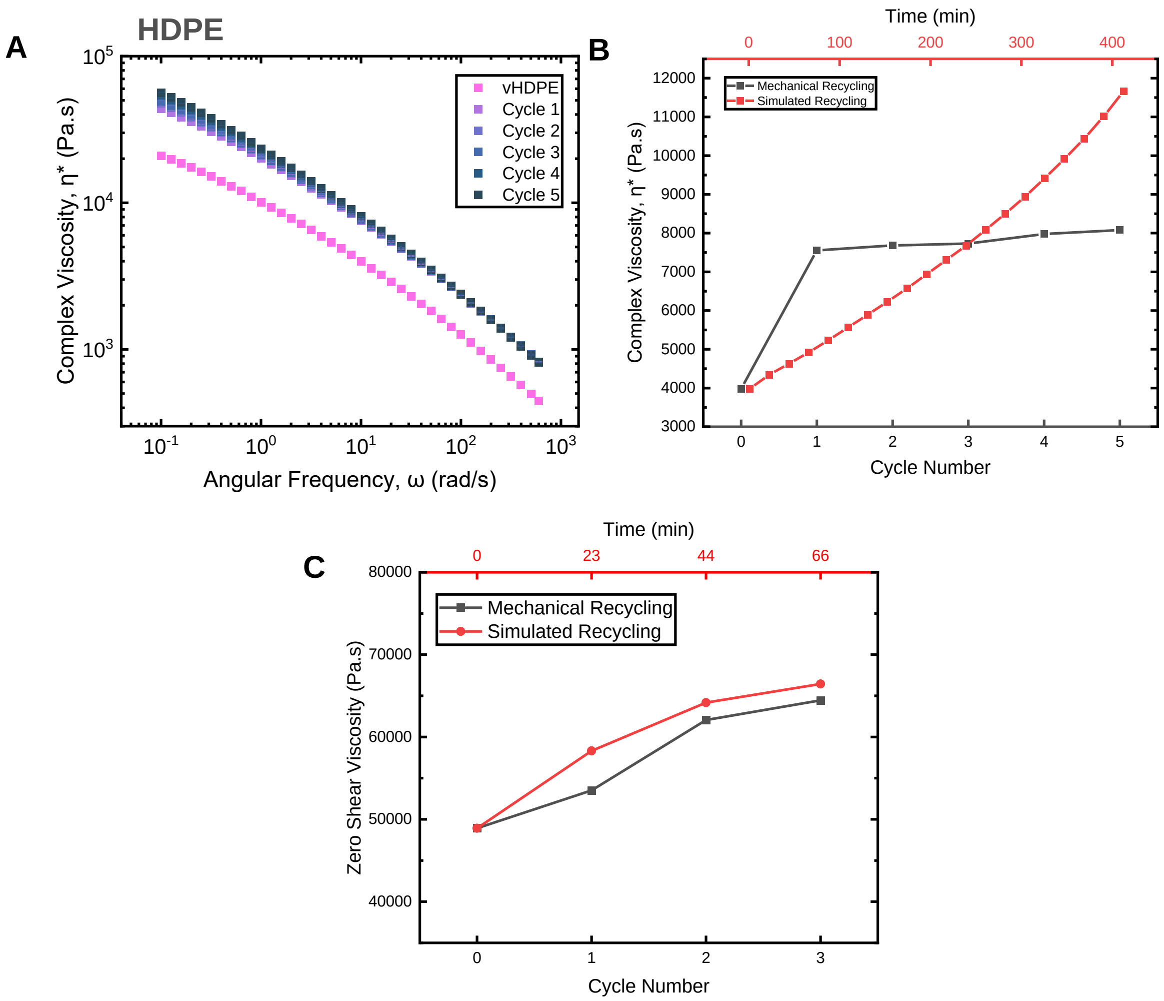

Figure S1 – (A) Frequency Sweeps of HDPE samples after mechanical recycling from virgin up to 5 recycles, (B) their mapping to rheological simulated recycling at 10 rad s^{-1}, HDPE and (C) Zero Shear Viscosity as calculated by Carreau-Yasuda regression. Samples measured at 200 ˚C and set strain (HDPE: 0.3%). Simulated recycling measured with frequency sweeps as a function of time (Red). Post-extrusion frequency sweeps are measured as a function of mechanical recycling cycles (Black). Extrusion cycle frequency sweeps measured in N_2 to mitigate further degradation whereas rheological simulation occurs in air. Standard error calculated from triplicate measurements.

From frequency sweeps on samples produced from traditional mechanical recycling methods, performed in a twin-screw extruder (A), we see a substantial jump in complex viscosity (η*), before a more gradual increase per cycle (***Figure S1***). In a melt extruder the first extrusion cycle is shown to have a significant impact on the polymer, occurring from the mechanical shear force applied to the polymer from the 8 to O transition within the extruder, with the degradation observed on first processing correlating to approximately 200 minutes in vGP tests. Zero shear viscosity was calculated via Carreau-Yasuda regression to determine how representative mechanical recycling was to our simulated method.

Good correlation was found between zero shear viscosity of both simulated and mechanical recycling methods, suggesting that fundamental polymer changes are similar in both methods (***Figure S1C***).

Additive packages as well as a broad molecular weight distribution (Đ > 7 as measured with HT-SEC) for the virgin polymer, used in food packaging, contribute significantly to the jump in η* within the initial extrusion before this effect is normalised after subsequent extrusions. The Cox-Merz rule states that complex viscosity as a function of frequency (as measured during oscillatory rheology) is equivalent to steady shear viscosity as a function of shear rate (as within a continuous co-rotating extruder), explaining their equivalence. This rule holds for simple solutions and polymer melts, however more complex dispersions can show variation.

2) Crossover Modulus

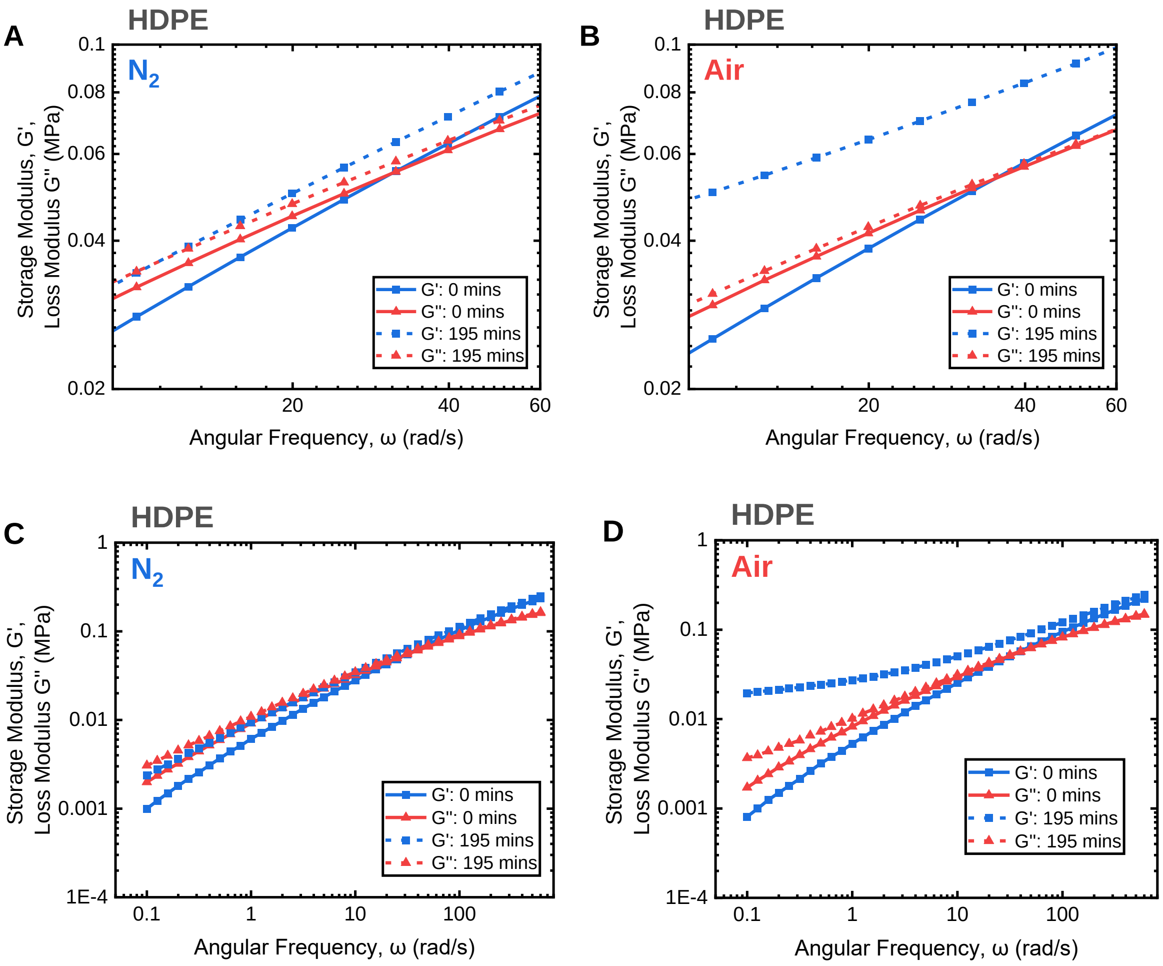

Figure S2 – (A) Modulus crossover points of HDPE treated in N_2, and (B) modulus crossover points of HDPE treated in air before and after time-resolved rheology. Plots (C) and (D) show a full-scaled axis for plots (A) and (B) respectively – for the clarity of determination of crossover points. Note: Figure S2B does not have a modulus crossover point after treatment.

In these plots, the crossover modulus is indicated by the point at which G' = G'' for both pre- and post-simulated extrusion. As the crossover modulus moves from left to right (x-axis) the molecular weight decreases and from top to bottom (y-axis) the molecular weight distribution broadens. In ***Figure S2A*** (treatment in N_2) the crossover point is shifting left across the x-axis (towards low angular frequencies) and down across the y-axis (towards lower moduli) indicating a higher molecular weight with a broader molecular weight distribution (vs vHDPE). When G' > G'' and there is not a crossover modulus, it is implied there is a covalent network, which in this case is indicative of extensive chain branching within polymer treated in air (***Figure S2B***).

3) Gas-Mediated Simulated Recycling of polyolefins in gaseous environments.

Altering the gaseous environment when HDPE is under thermo-mechanical shear produces significant changes in the chemical structure of the polymer and can be linked to the presence of O_2 in air. Varying gaseous environment to alternative inert gases (CO_2, Argon – ***Figure S5, S6***) produced no significant viscoelastic impact upon testing in comparison to N_2.

Changing the composition of the gaseous environment during gas-mediated simulated recycling was performed to further elucidate the impact of O_2 on degradation mechanisms. Mixtures of N_2 and air were used in ratios increasing in increments of 25% (ranging from 100% to 0% N_2:air). ***Figure S7*** outlines the vGP plots of these gas mixtures. A gradual change in the left flank of the vGP curve proportional to increasing O_2 in the environment was observed. With increasing O_2 content there is an increasing incidence of long chain branching (LCB) — evidenced by shifting of vGP traces to lower phase angle and lower complex modulus values — consistent with previous measurements.

The presence of inert gas has a blanketing effect on the polymer melt, limiting the formation of peroxyl radicals which accelerate the degradation of polyolefins. Increasing the flow rate of gas (< 5 L min^{-1}) led to high rates of change of viscosity, signifying a flow rate reactivity dependence (***Figure S8***) with relative changes in complex viscosity being measured versus initial viscosity (***Equation S1, S2***). This gas dependence can be attributed to two factors: 1) the ability of gas to flow throughout the environmental test chamber, and 2) the formation of hot spots within the polymer melts at low flows, increasing degradation across the sample. As such, experiments were performed at a set flow of 15 L min^{-1} to standardise the impact of gas flow.

Testing time was increased by doubling the number of sequential frequency sweeps to 20 to identify a high-end limit for LCB (***Figure S9***). It was found that no change in viscosity was seen after 218 minutes for HDPE treated under N_2, possibly indicating a limit to thermo-mechanical degradation. This limit was reflected by a plateau in vGP curve shifts after 218 minutes of testing. Conversely, HDPE treated in air continues to undergo LCB, as demonstrated by substantial increases in complex viscosity, complex modulus, and the shape of the vGP curve approaching a 180° arc, indicative of a pure LCB component.

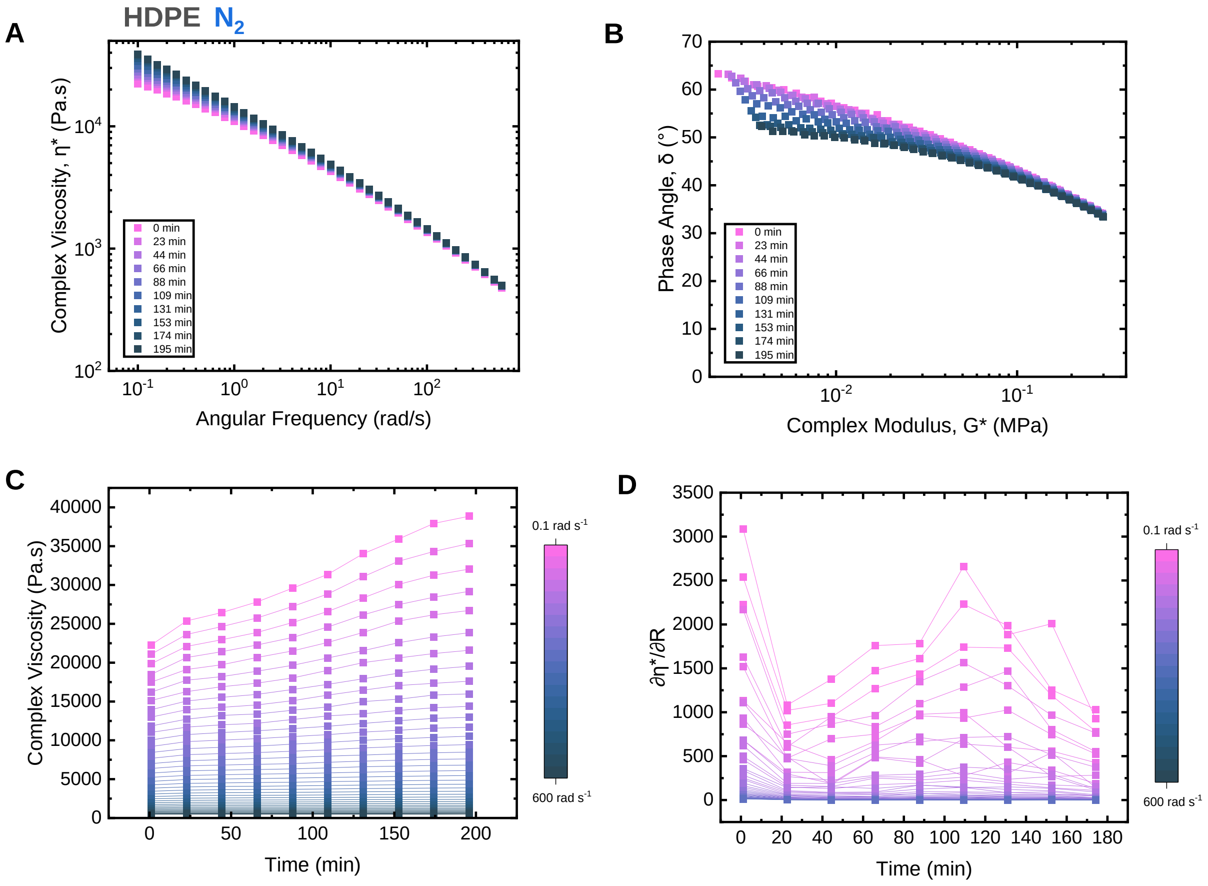

Figure S3 – Rheological recycling simulation of HDPE under N_2. (A) Complex viscosity extracted from sequential frequency sweeps of HDPE samples at 200 °C, from 0.1 to 600 rad·s^{-1} at 0.3 % strain with 12-minute intervals. **(B)** Van Gurp-Palmen plot of rheological recycling simulation determined from **(A)**. **(C)** Complex viscosity at different angular frequencies during sequential frequency sweeps with time. **(D)** Rate of change of complex viscosity during rheological recycling simulation, determined from **(C)**

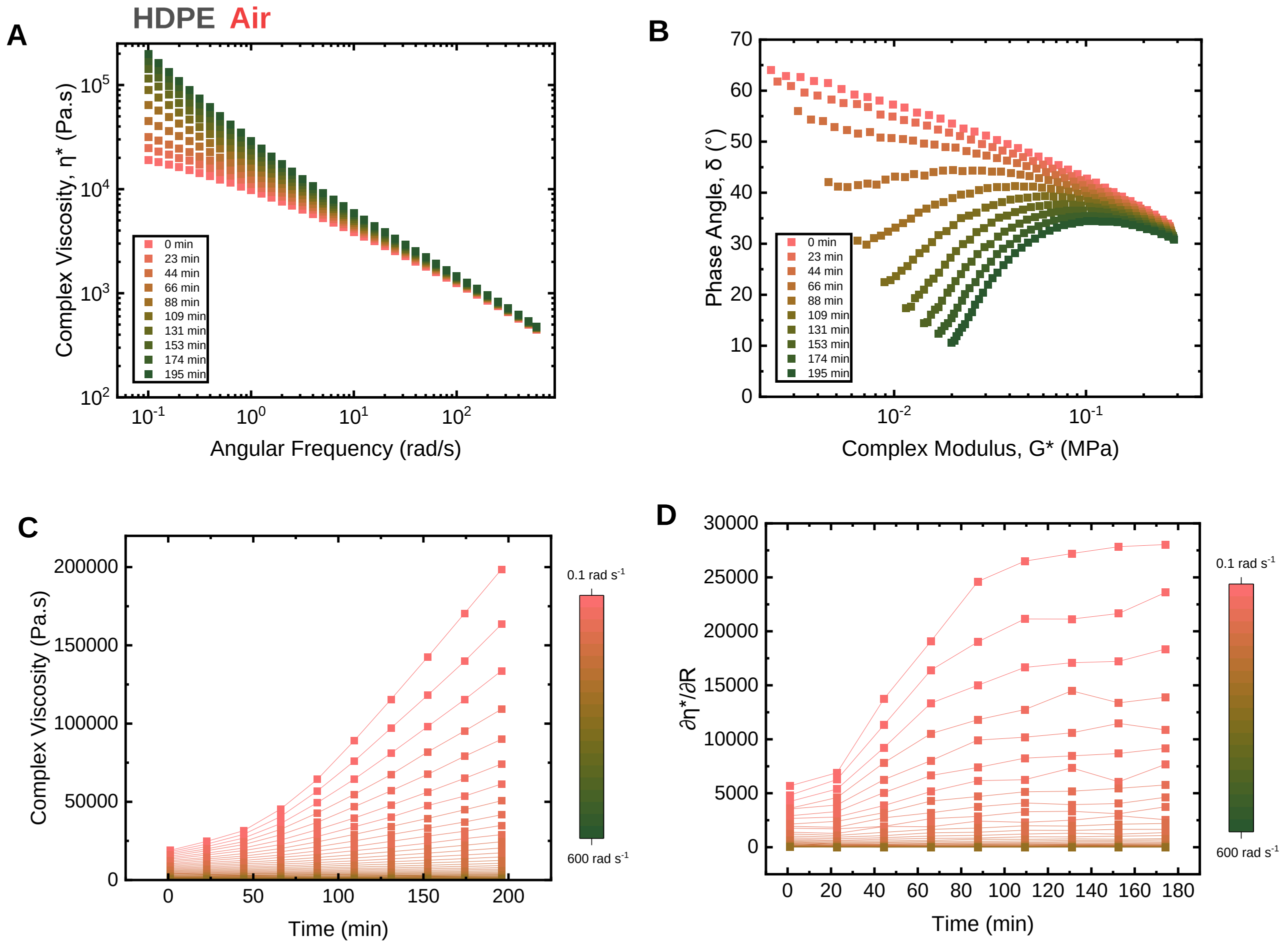

Figure S4 – Rheological recycling simulation of HDPE under Air. (A) Complex viscosity extracted from sequential frequency sweeps of HDPE samples at 200 °C, from 0.1 to 600 rad·s^{-1} at 0.3 % strain with 12-minute intervals. **(B)** Van Gurp-Palmen plot of rheological recycling simulation determined from **(A)**. **(C)** Complex viscosity at different angular frequencies during sequential frequency sweeps with time. **(D)** Rate of change of complex viscosity during rheological recycling simulation, determined from **(C)**.

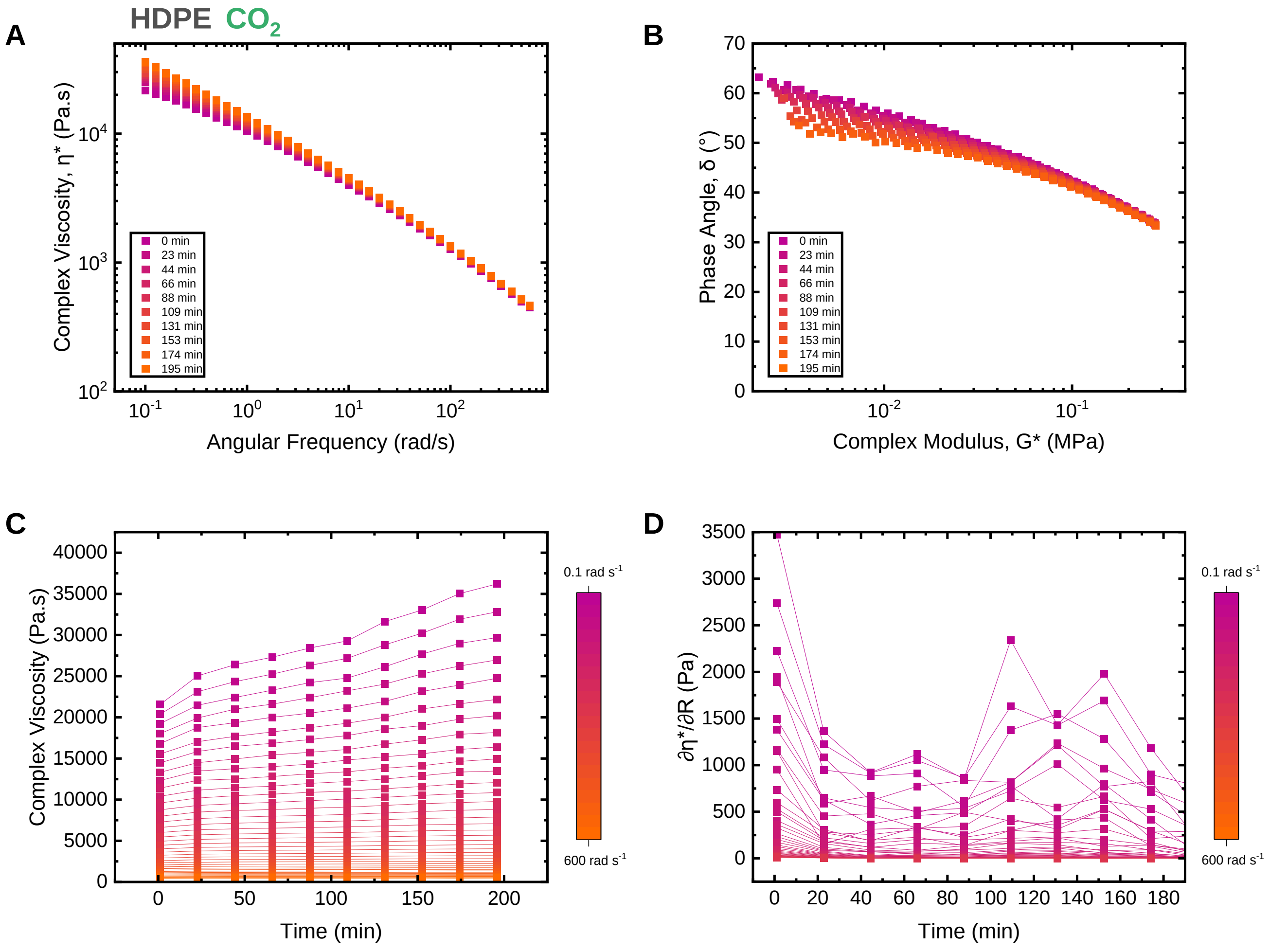

Figure S5 – Rheological recycling simulation of HDPE under CO_2. (A) Complex viscosity extracted from sequential frequency sweeps of HDPE samples at 200 °C, from 0.1 to 600 rad·s^{-1} at 0.3 % strain with 12-minute intervals. **(B)** Van Gurp-Palmen plot of rheological recycling simulation determined from **(A)**. **(C)** Complex viscosity at different angular frequencies during sequential frequency sweeps with time. **(D)** Rate of change of complex viscosity during rheological recycling simulation, determined from **(C)**.

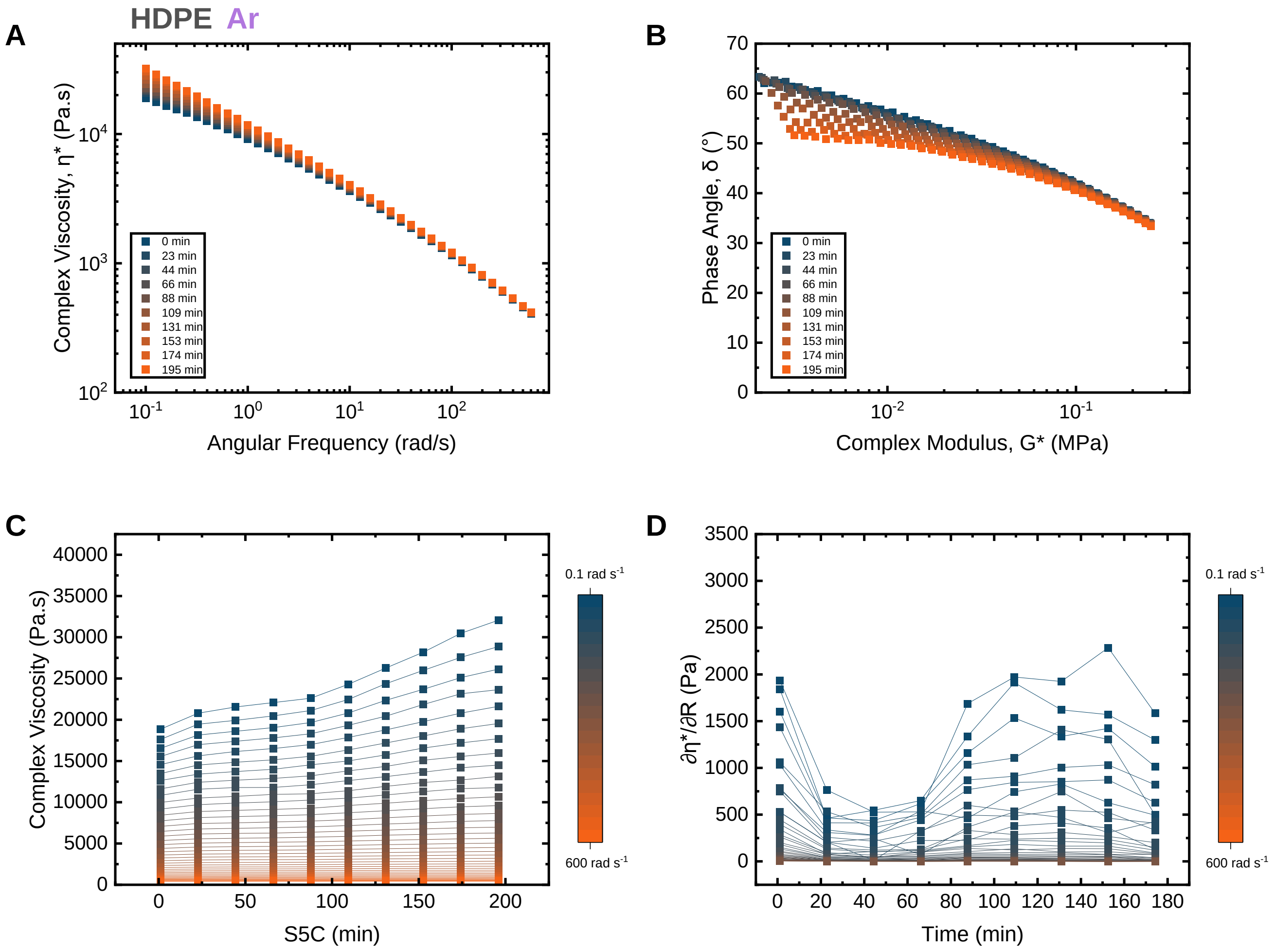

Figure S6 – Rheological recycling simulation of HDPE under Ar. (A) Complex viscosity extracted from sequential frequency sweeps of HDPE samples at 200 °C, from 0.1 to 600 rad·s^{-1} at 0.3 % strain with 12-minute intervals. **(B)** Van Gurp-Palmen plot of rheological recycling simulation determined from **(A)**. **(C)** Complex viscosity at different angular frequencies during sequential frequency sweeps with time. **(D)** Rate of change of complex viscosity during rheological recycling simulation, determined from **(C)**.

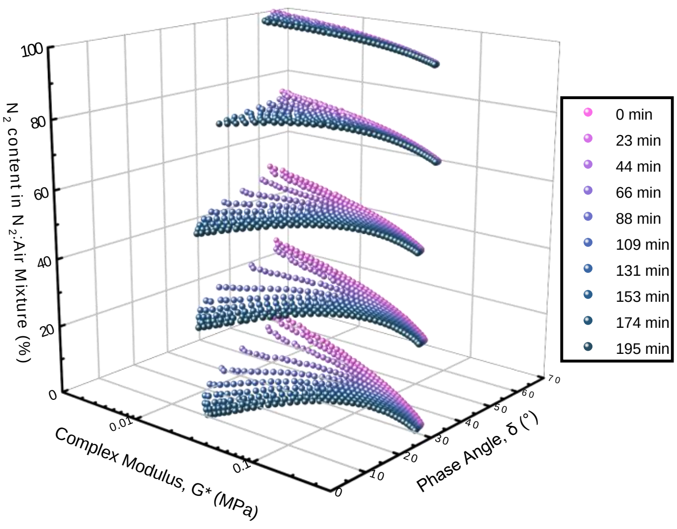

Figure S7 – Van Gurp-Palmen plots detailing the change in the degradation mechanism of HDPE during rheological recycling simulation under multiple N_2:Air mixtures. In order of N_2:Air ratios, 100:0 (Top), 75:25, 50:50, 25:75, 0:100 (Bottom). Plots extracted from complex viscosity at different angular frequencies during sequential frequency sweeps at 200 °C, from 0.1 to 600 rad·s^{-1} at 0.3 % strain with 12-minute intervals. There is a measurable effect of the increase of air within the gaseous environment, suggesting that the concentration of O_2 is proportional to the amount of degradation measured within HDPE.

4) Flow rate variation

Samples underwent frequency sweeps before and after the 3-hour time sweep experiment. The relative change in the complex viscosity was determined with the following two equations:

$$\Delta\eta_R^* = \frac{\eta_i^*}{\eta_0^*} \qquad (\boldsymbol{S1})$$

$$\Delta\eta_t^* = \eta_i^* - \eta_0^* \qquad (\boldsymbol{S2})$$

Where:

$\boldsymbol{\eta_0^*}$ is the initial complex viscosity at time t = 0.

$\boldsymbol{\eta_i^*}$ is the complex viscosity at a given point of time,

$\boldsymbol{\Delta\eta_t^*}$ is the change in complex viscosity,

$\boldsymbol{\Delta\eta_R^*}$ is the change in complex viscosity relative to the initial viscosity,

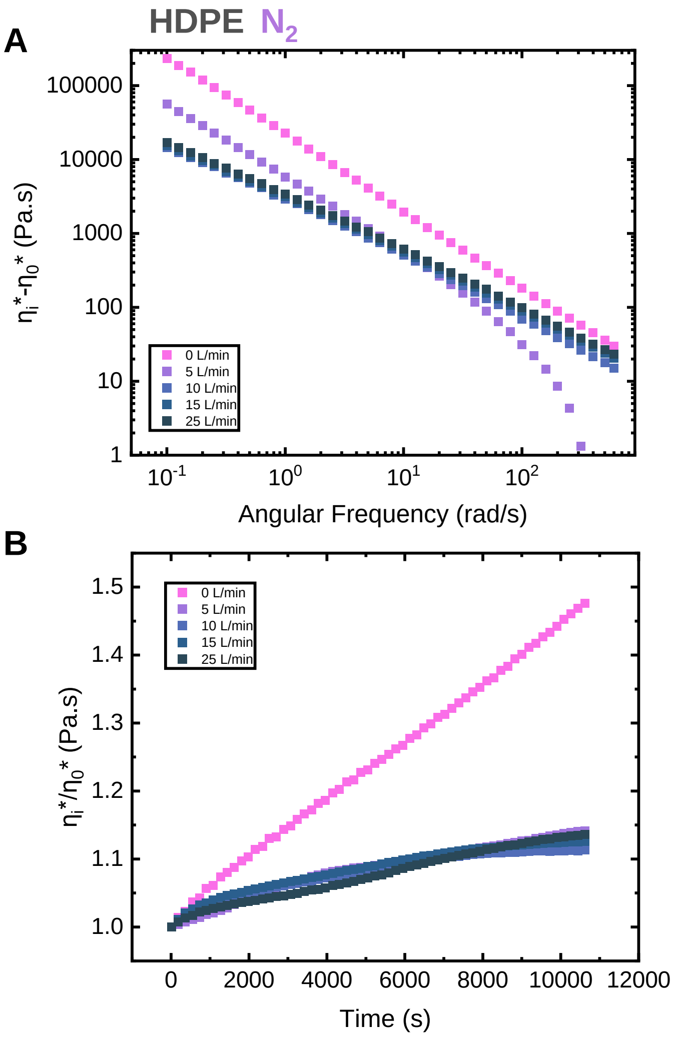

Figure S8 – Analysis of flow rate post time sweep of HDPE treated in N_2. (A) The change in complex viscosity after time sweep. **(B)** The ratio of complex viscosity versus initial viscosity value with time. Identical frequency sweeps at 200 °C, from 0.1 to 600 rad·s^{-1} at 0.3 % strain.

Variation of flow rate resulted in a significant impact on the change in complex viscosity during a time sweep. We hypothesise that the incidence of increased gas flow around the polymer melt allowed for a blanketing effect, preventing radical generation and permeation throughout the polymer melt. Of note is the formation of hot spots across the surface of the geometry when there is no gas flow to the environmental test chamber, which is recognised as an instrumental limitation. The test chamber allows for an inlet of gas which flows through convection. Without gas flow, heat is not evenly distributed within the chamber, which leads to additional degradation and is evidenced by the significant increase in the ratios of complex viscosity (***Figure S7)***.

5) Degradation limit determination for HDPE

Limits of HDPE degradation were determined by extended gas-simulated rheological experiments. HDPE underwent 20 consecutive frequency sweeps, each separated by isothermal holds (no oscillation) of 12 minutes.

By extending testing time from 195 to 412 minutes we aimed to ensure prolonged degradation within the polymer melt. The rate and change of degradation mechanism in N_2 stop at 218 minutes, suggesting that there is no additional chain branching within an inert environment after this point (***Figure S8A***). This plateau evidences the benefit of mechanically recycling under an inert environment and could make polyethylene significantly more processable industrially. Within air, there is continuous chain branching until a pure long-chain branching component is created (***Figure S8B***). **Figure S9** outlines the endpoints of this change in the vGP plot, suggesting that this material will operate within an initial and final boundary when mechanically recycled. This knowledge could allow for the use of a process controller within the mechanical recycling process once the polymer melt reaches a limit. Here we can use the limit taken as the point at which the degradation of HDPE in N_2 stops changing (218 minutes), to state that any material that has a curvature beyond this limit is not fit for processing. This statement is only used as an example, however, as a vGP plot is temperature, polymer grade, and process history independent. We can use extensively tested post-consumer recyclate to further determine limits bespoke to industrial recyclate.

A

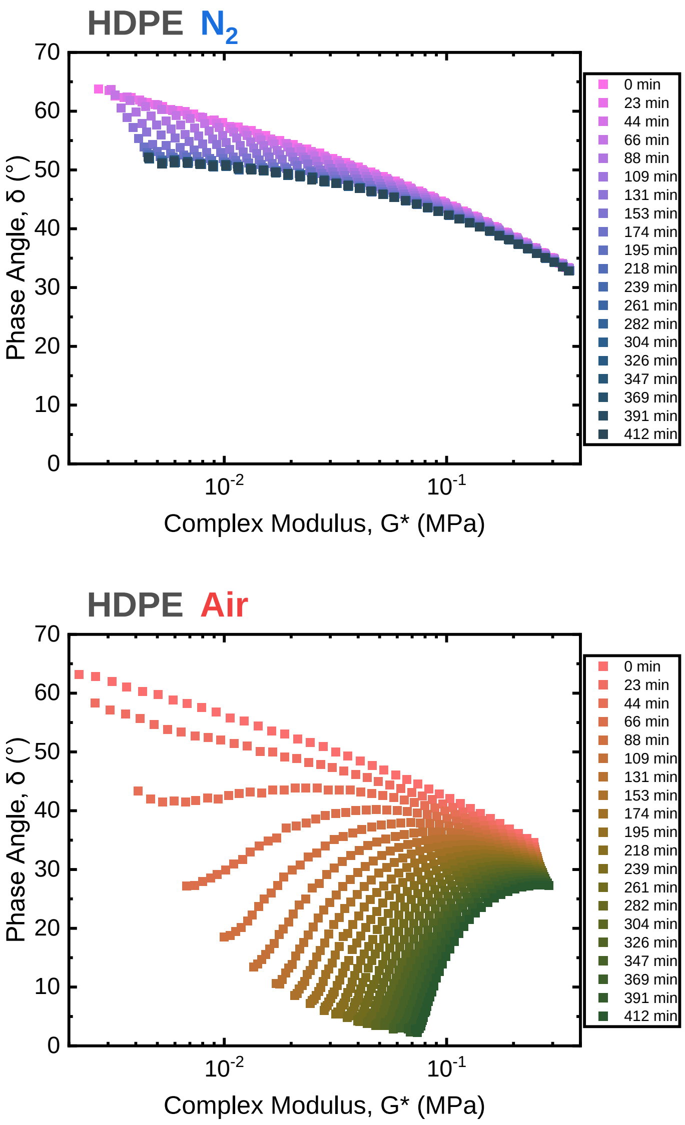

B

Figure S9 – Extended rheological recycling simulation of HDPE under N_2 and air. Van Gurp-Palmen plots of rheological recycling simulation extracted from sequential frequency sweeps of HDPE samples at 200 °C, from 0.1 to 600 rad·s^{-1} at 0.3 % strain with 12-minute intervals over 412 minutes in **(A)** N_2 and **(B)** air.

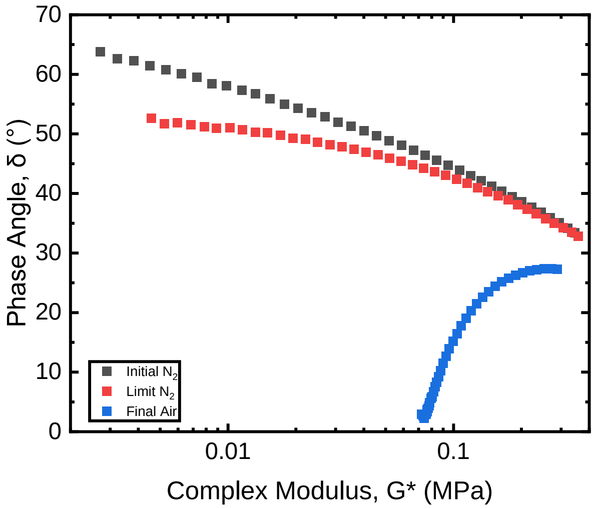

Figure S10 – Limit setting of Van Gurp-Palmen plot to determine maximum and minimum limits of HDPE degradation in mechanical recycling. Plot extracted from Figure S8. Initial N_2 extracted from the first frequency sweep of Figure S8A. Limit N_2 taken from the point at which vGP stops changing in Figure S8A, t = 218 min. Final air is taken from *Figure S8B* at t = 412 min.

6) Rheological Dependence of Van Gurp-Palmen Plot

$$|G^*| = \sigma_0/\gamma_0 \qquad (\boldsymbol{S3})$$

Where:

$|\boldsymbol{G}^*|$ is the magnitude of the complex modulus.

σ_0 is the amplitude of oscillatory stress.

γ_0 is the amplitude of oscillatory shear.

$$G^* = G' + iG'' \qquad (\boldsymbol{S4})$$

$$\log\ G'' = \log\ \eta_0 + \log\ \omega \qquad (\boldsymbol{S5})$$

$$\log\ G' = \log\ \eta_0^2\ J_e^0 + 2\ \log\ \omega \qquad (\boldsymbol{S6})$$

Where:

$\boldsymbol{G}^*$ is the complex modulus composed of a real part $\boldsymbol{G}'$ and an imaginary part $\boldsymbol{G}''$.

η_0 is the zero-shear viscosity.

ω is the measured angular frequency.

J_e^0 is the linear steady-state recoverable compliance.

$$[\eta] = KM^a \qquad (\boldsymbol{S7})$$

$$\eta_0 = a(M/M_c)^a \text{ for } M \geq M_c$$
$$\eta_0 = a(M/M_c) \text{ for } M \leq M_c \qquad (\boldsymbol{S8})$$

Where:

$[\eta]$ is the intrinsic viscosity of a given macromolecule.

η_0 is the zero shear viscosity for a given polymer melt.

K a solvent- polymer dependent fitting parameter.

M is the molar mass.

M_c is the critical molar mass molar mass.

a is a solvent-polymer dependant fitting parameter ≅ 3.4-3.6 and is a constant dependant on the type of polymer and the temperature.

The complex modulus can be directly calculated from frequency sweeps (complex viscosity vs angular frequency), providing a quantitative measure of resistance to deformation (***Equation S3***). The difference between the elastic and viscous components of the polymer melt (i.e., the phase difference) allows for a relative composition of a polymer to be determined, the phase angle is a relative measure of viscous and elastic characteristics.

The complex modulus is composed of two parts, the real and imaginary components (***Equation S4***). The steady-state recoverable compliance, J_e^0, is associated with the elastic component of the polymer, and is directly linked to the zero-shear viscosity, η_0, through the components of the complex moduli (***Equation S5, S6***). Thus, this explains the empirical relationship between molar mass and the viscosity of linear polymer melts (***Equation S7***), with the zero-shear viscosity being directly proportional to molar mass above a specific critical molas mass (***Equation S8***).

As a vGP plot is a plot of complex modulus versus phase angle, there is enough information within the plot to extract compositional features, directly related to the molar mass and zero shear viscosity, which can rapidly allow for a relative quality determination.

7) Qualification of V_{deg} in linear polymer melts

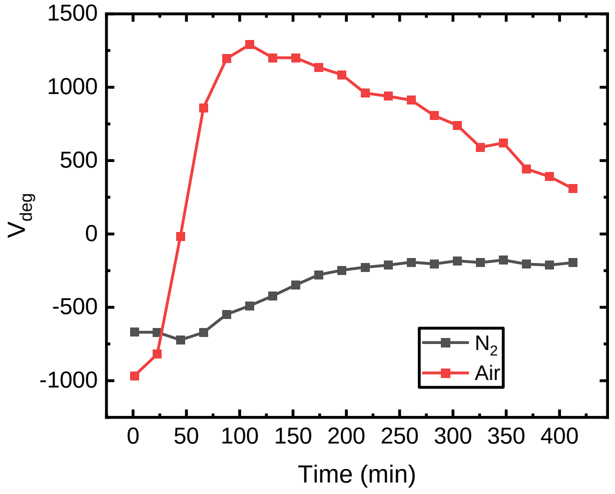

Figure S11 – Plot of V_{deg} versus time for HDPE under N_2 and air during extended rheological simulated recycling. Measurements taken at 200 °C, from 0.1 to 600 rad·s^{-1} at 0.3 % strain with 12-minute intervals.

8) Mixed gases apparatus

Gases were mixed using parallel flow controllers connected to the laboratory's gas supply. Tubing was connected to supply for two gases, A and B, and featured an extended section that served as a mixing zone. This design enhanced the mixing efficiency of the gases. The parallel flow controllers then fed the gas mixture into the environmental test chamber of the rheometer through a push-fit Y-Connector, where the gas was drawn in at a fixed rate of 15 L min^{-1}. The mixing ratios of Gas A to Gas B were modulated as required. This apparatus was also used and outlined in previous work.

9) Extrusion of polyolefins under gaseous environments

Extrusion experiments were conducted in a HAAKE Minilab micro compounder at 100 rpm, 200 °C under 1 bar purge gas in flush mode. Premixed gases were supplied through an external cylinder.

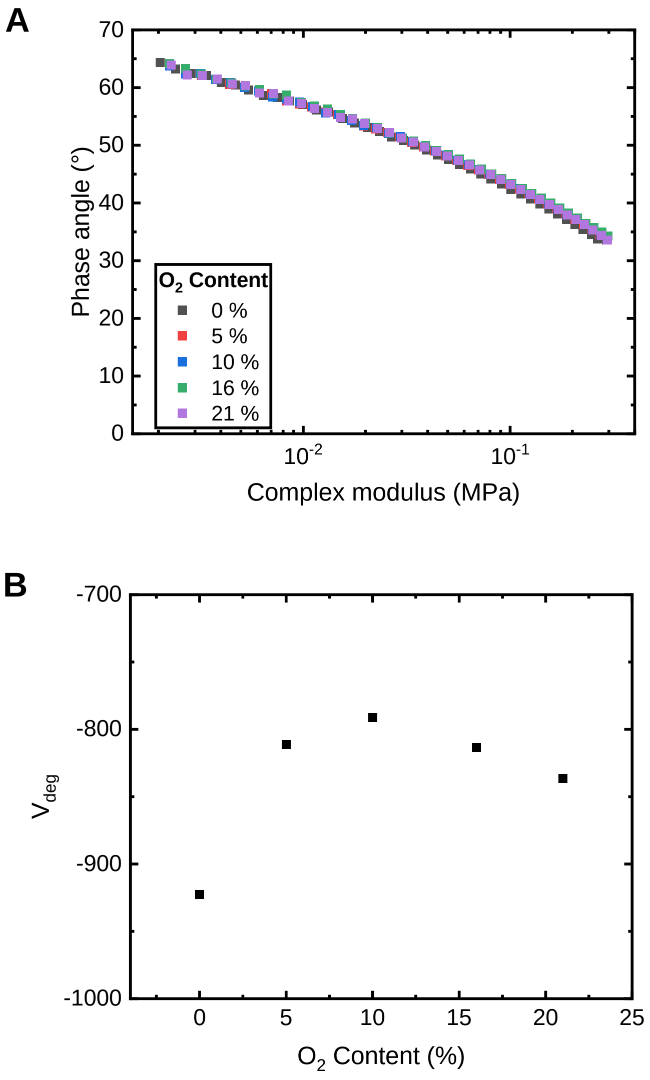

Figure S12 – Analysis of HDPE after extrusion under different gaseous environments of increasing O_2. (A) Van Gurp-Palmen plots for extruded material. **(B)** V_{deg} extracted from **(A)** for extruded material. Measurements taken at 200 °C, from 0.1 to 600 rad·s^{-1} at 0.3 % strain with 12-minute intervals.

Variation in the Van-Gurp Palmen plot is limited when extruded in a twin-screw micro-compounder. It is important to outline the scale of the equipment used when discussing the results, and understanding how meaningful they are in the context of PE. First, there are safety considerations to consider: using higher concentrations of O_2 to exacerbate degradation and using a larger sealed environment around the micro-compounder is possible; however, it is costly, time-consuming, and unsafe due to the nature of high O_2-content gases. Experimental adaptations were made to run the polymer under purge, including using Air:N_2 mixtures as opposed to O_2:N_2 mixtures, and scaling the experiment down to resolve safety concerns.

During the experiment, the polymer was processed continuously through a relatively small, twin-conical screw barrel, meaning the time in the melt was relatively short before being extruded (<1 min). However, there is measurable variation in V_{deg} with extrudate under an inert N_2 atmosphere and atmospheres with O_2 present (***Figure S11B***). HDPE can be processed extensively, with initial changes being lesser than that of PET which can readily degrade. Previous work outlines the impact of the same processing conditions and apparatus on PET.

10) Simulated extrusion of differing grades of HDPE

A range of polymers were selected for use in testing: three virgin polymers or similar melt flows suitable for blow moulding, injection moulding, packaging, and food purposes. These were chosen primarily for their current potential use cases in packaging and are closely related to commonly manufactured products (FMCGs), with the overall aim being to simulate real-world extrusion conditions.

Multiple grades of PCR were used, originating from multiple sources around the world. Upon initial investigation these materials had similar measured melt flow rates (MFRs), crystallinities and T_{deg} 5%; however, were visually, mechanically, and chemically (M_w) distinct. Different grades were assigned to polymers provided by suppliers, largely falling into three categories, virgin HDPE, natural grade recyclate, and jazz grade recyclate.

As PCR is inherently variable, simulated extrusions were performed in triplicate, with an example of error and deviation outlined below. For clarity of figures, error bars have not been overlaid on further plots.

Generally, the mechanism of degradation shown in all samples corresponds to that of the single grade of vHDPE used in initial experiments. (***Figure S12***) The rate of degradation and the extent to which a particular polymer does degrade is the key differentiator within grades of HDPE. The methodology is reproducible with a low incidence of standard error even when accounting for industrially sourced PCR (***Figure S13***). As expected, specific grades of HDPE behaved similarly when tested, with vHDPE degrading to a great extent, but not significantly chain branching until extensive amounts of processing (t >66 mins) (***Figure S14***). Various sources of natural grade HDPE show similar behaviour to each other. PCR 2 is dyed white to obfuscate any visual contaminants, yet degrades at a much faster rate, despite being a natural grade PCR (***Figure S15***). The jazz grade PCRs undergo the most significant degradation, with extensive chain branching occurring rapidly (***Figure S16***).

Normalisation of the raw V_{deg} data enables a complicated methodology to be easily visualised; however, there are caveats to the process of normalisation which can alter the interpretation of results qualitatively. This raw, unnormalized data provides the most complete picture in terms of quality and composition, but it is cluttered and unclear. Applying it to a process control gate would be complicated and inefficient. As a vGP plot is temperature, grade, and process independent, we can normalise data to extract information rapidly. Normalising each vGP trace to [0, 100] allows for an easily compared picture of the rate of degradation of the polymer but assumes the limits of degradation of HDPE are uniform across grades. As all grades of HDPE commonly used in manufacturing have additive packages which help with material performance, and recyclate more so, the normalisation process can be inappropriate for particular use cases. Further, normalisation to a minimum is functionally appropriate, but assumes that each polymer is functionally equal which is often not the case between grades. However, this may be a way to determine the differences in material performance of like-materials. Normalising vGPs to [0, 100] has been selected as an exemplar in the main body of text due to its relative ease of understanding and its applicability to industry.

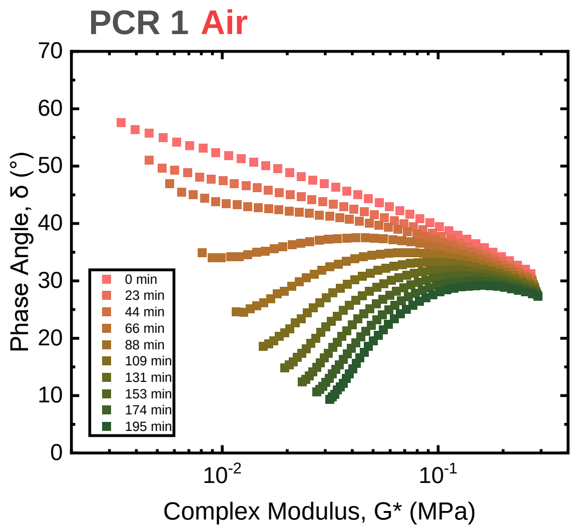

Figure S13 – Rheological recycling simulation of PCR 1 under air produced as an exemplar. Van Gurp-Palmen plot of rheological recycling simulation determined from successive frequency sweeps. Van Gurp-Palmen plots of rheological recycling simulation extracted from sequential frequency sweeps of HDPE samples at 200 °C, from 0.1 to 600 rad·s-1 at 0.3 % strain with 12-minute intervals over 195 minutes in air.

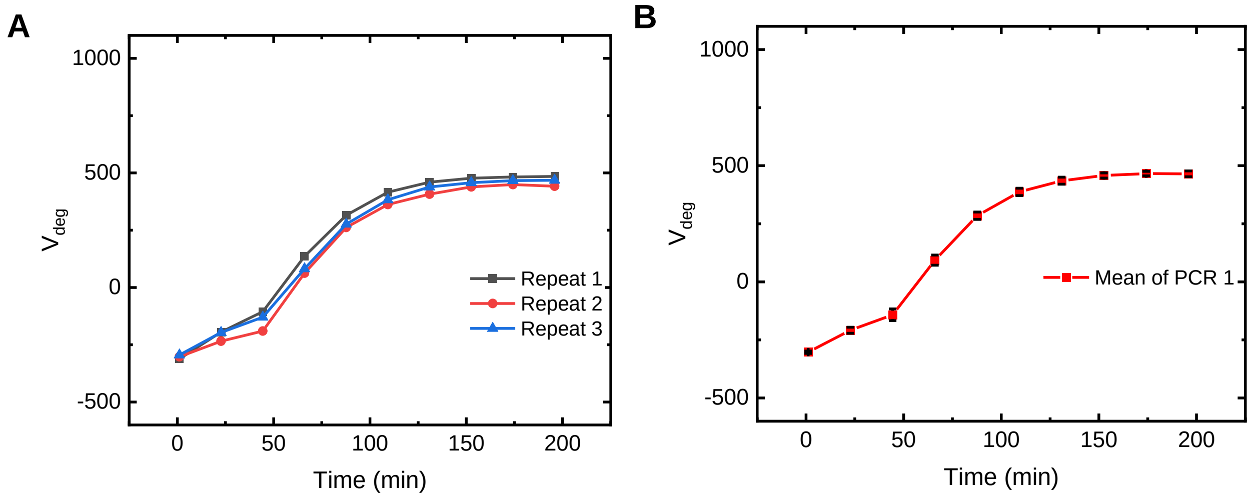

Figure S14 – (A) Plot of V_{deg} versus time for PCR 1 under air during rheological simulated recycling, produced in triplicate and (B) and mean plot of V_{deg} versus time for PCR 1 under air during rheological simulated recycling with standard error. Measurements taken at 200 °C, from 0.1 to 600 rad·s^{-1} at 0.3 % strain with 12-minute intervals.

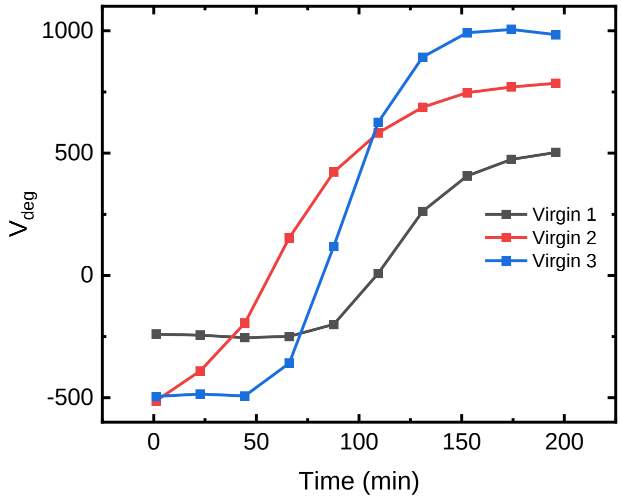

Figure S15 – Plot of V_{deg} versus time for vHDPEs under air during rheological simulated recycling. Using raw data. Measurements taken at 200 °C, from 0.1 to 600 rad·s^{-1} at 0.3 % strain with 12-minute intervals.

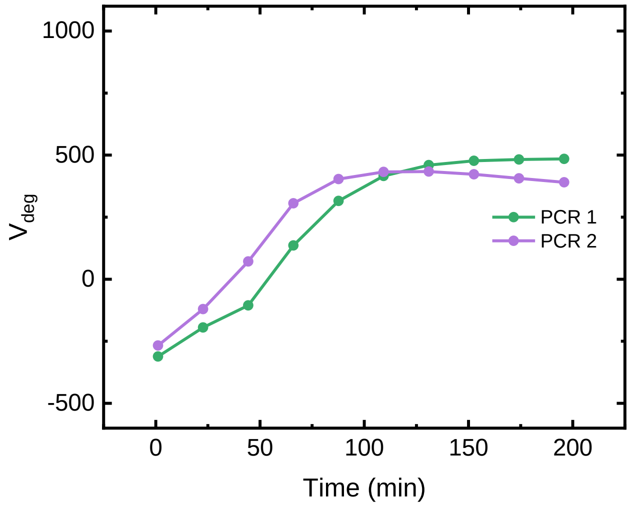

Figure S16 – Plot of V_{deg} versus time for natural grade PCR(HDPE)s under air during rheological simulated recycling. Using raw data. Measurements taken at 200 °C, from 0.1 to 600 rad·s^{-1} at 0.3 % strain with 12-minute intervals.

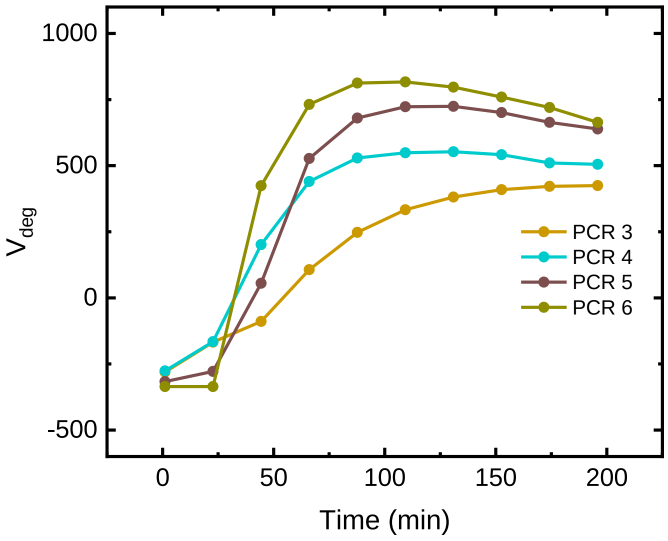

Figure S17 – Plot of V_{deg} versus time for jazz grade PCR(HDPE)s under air during rheological simulated recycling. Using raw data. Measurements taken at 200 °C, from 0.1 to 600 rad·s^{-1} at 0.3 % strain with 12-minute intervals.

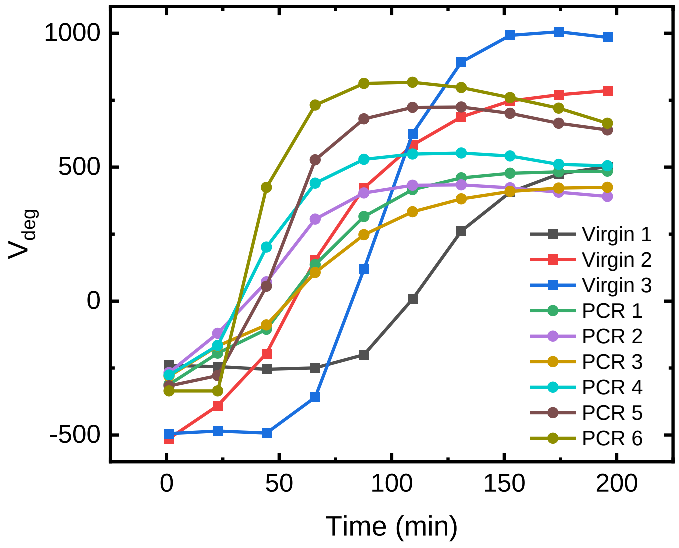

Figure S18 – Plot of V_{deg} versus time for multiple vHDPE and PCR(HDPE) under air during rheological simulated recycling. Using overlaid raw data. Measurements taken at 200 °C, from 0.1 to 600 rad·s^{-1} at 0.3 % strain with 12-minute intervals.

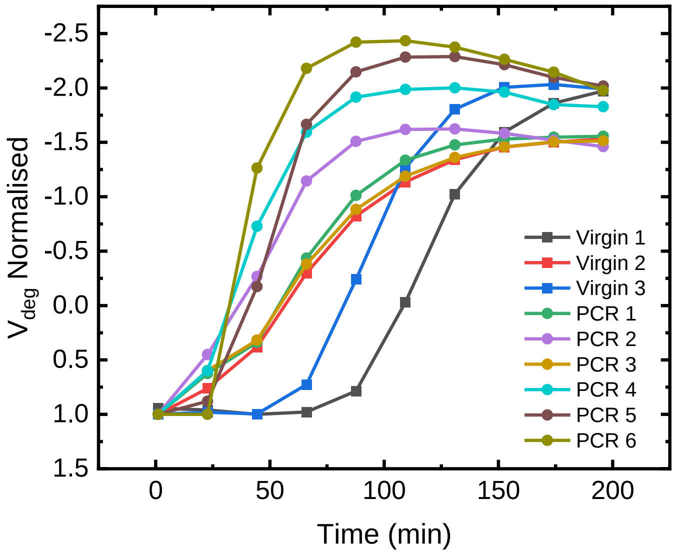

Figure S19 – Plot of V_{deg} versus time for multiple vHDPE and PCR(HDPE) under air during rheological simulated recycling. Normalised to a minimum value of raw data. Measurements taken at 200 °C, from 0.1 to 600 rad·s^{-1} at 0.3 % strain with 12-minute intervals.

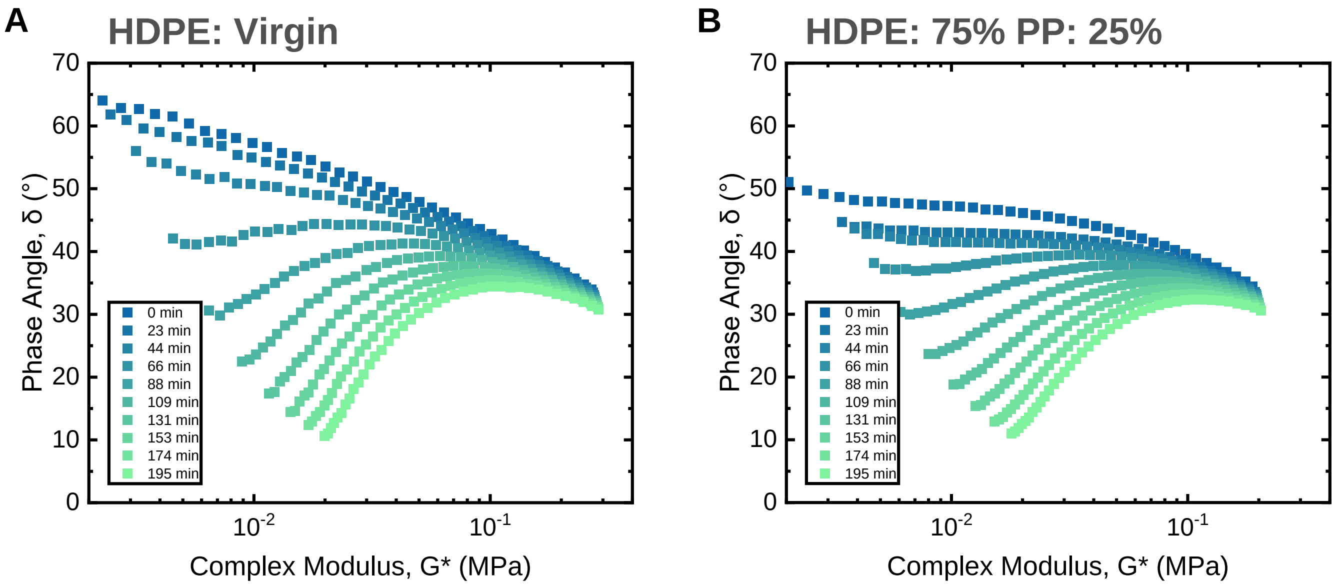

Figure S20 – Rheological recycling simulation of Virgin HDPE (0% PP) (A) versus HDPE/PP Blend (25% PP) (B) under air. Van Gurp-Palmen plots of rheological recycling simulation extracted from sequential frequency sweeps of HDPE and HDPE/PP samples at 200 °C, from 0.1 to 600 rad·s-1 at 0.3 % strain with 12-minute intervals over 195 minutes in air.

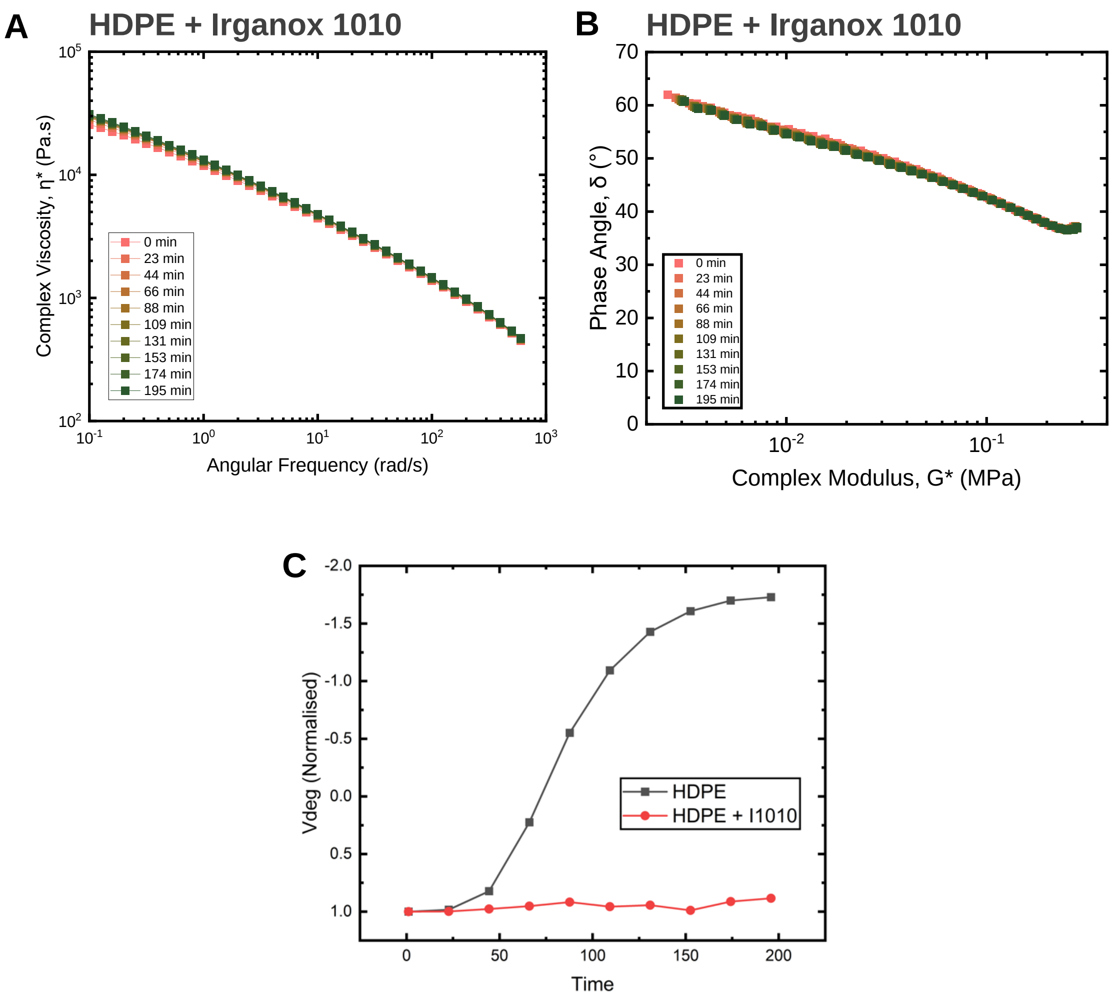

Figure S21 – Rheological recycling simulation of the impact of stabilisation systems within HDPE. Plots of complex viscosity versus angular frequency (A) and Van Gurp-Palmen plots for HDPE + 1% Irganox 1010 (B). Derived V_{deg} of HDPE/Irganox in comparison to vHDPE (C).

Coextrusion of Irganox 1010 (I1010), a commercial phenol-based primary antioxidant, with virgin HDPE was conducted using 1% I1010, the highest recommended loading to exacerbate a response to thermo-oxidative degradation. The addition of this antioxidant mitigates thermo-oxidative degradation in virgin HDPE, as reflected within both the changes to complex viscosity and delay in the change of V_{deg} as compared to its virgin PE equivalent. The additives stabilise radicals generated via hydrogen abstraction and mitigate a long chain branching degradation mechanism. This is reflected in the difference in Vdeg (**Figure S21**) showing a vHDPE substantially increasing V_{deg} after 50 minutes of processing, vHDPE+ I1010 showing minimal changes within the timeframe of the experiment.

11) Linear viscoelastic region determination and reproducibility within Van Gurp-Palmen plots

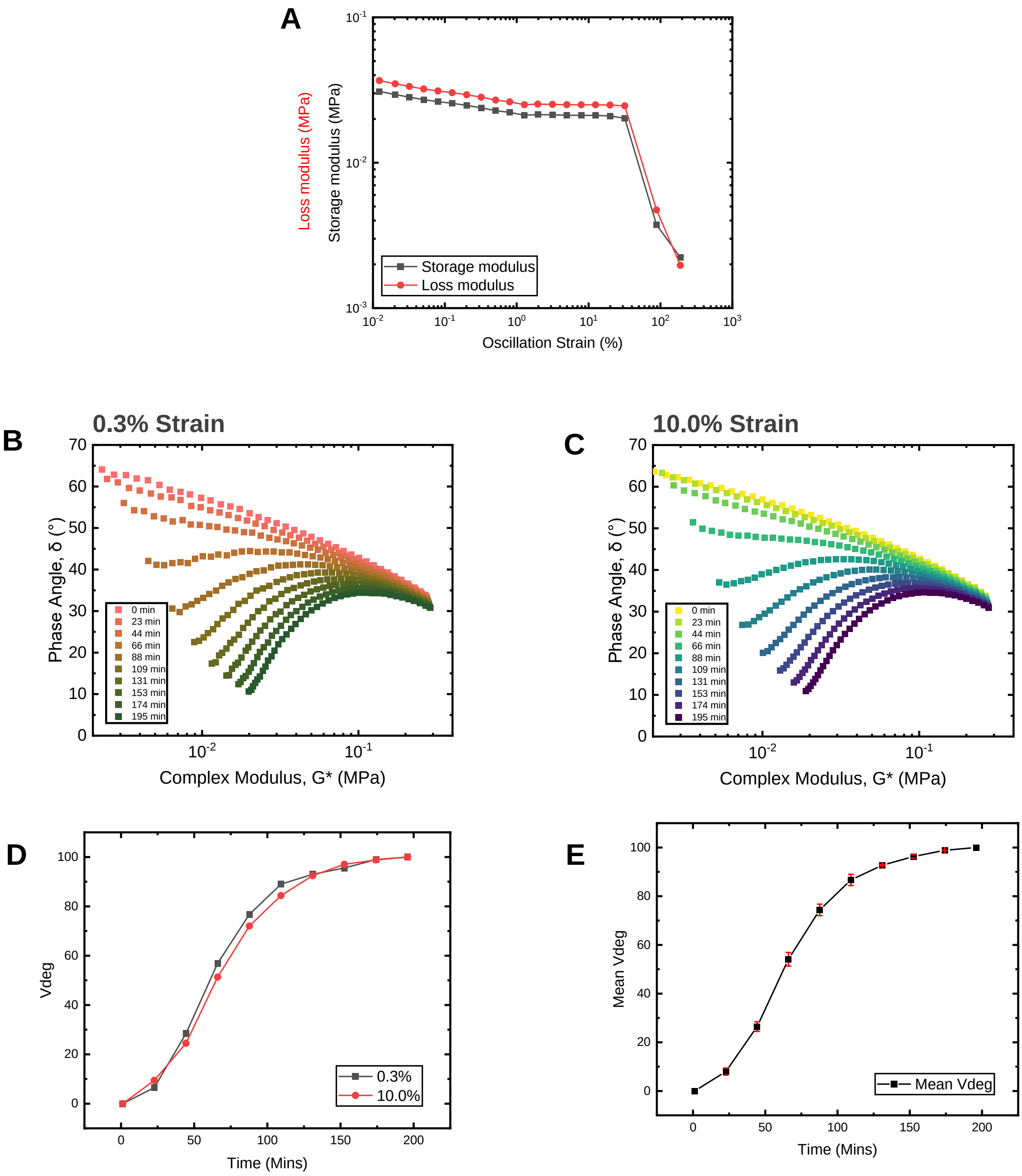

Figure S22 – Linear viscoelastic region determination of frequency sweeps by (A) Oscillatory amplitude sweep and rheological recycling simulation of HDPE with (B) 0.3% strain and (C) 10.0% strain. Van Gurp-Palmen plots of rheological recycling simulation extracted from sequential frequency sweeps of HDPE samples at 200 °C, from 0.1 to 600 rad·s^{-1} with 12-minute intervals over 412 minutes in air. Extracted Vdeg of plots for comparison showing differences in both strain amplitudes (D) and standard error between sweeps (E).

Oscillatory amplitude sweeps (10 rad·s^{-1}, 0.1–100 % strain, 200 °C) were performed to determine the testing strain falling within the viscoelastic region of the polymer melt (0.3 % HDPE, 1.0 % PP). There was minimal change found in data between extremes of the linear viscoelastic region, equivalent to degree of variance found in testing at a fixed strain (section 10 of supplementary information).

12) Time-temperature superposition of Van Gurp-Palmen plots

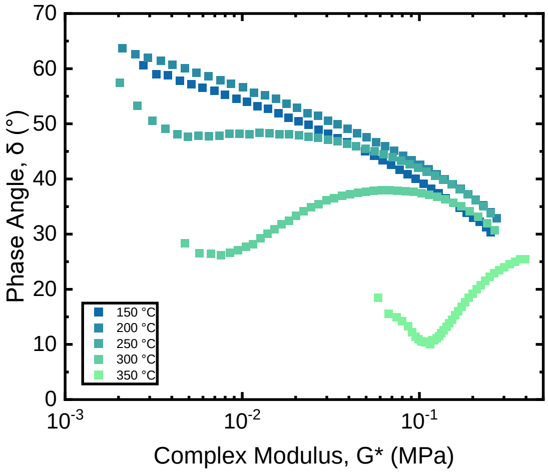

Figure S23 – Rheological recycling simulation of Virgin HDPE at a range of temperature – 150 °C to 350 °C. Van Gurp-Palmen plot of rheological recycling simulation determined from successive frequency sweeps. Van Gurp-Palmen plots of rheological recycling simulation extracted from sequential frequency sweeps of HDPE samples, from 0.1 to 600 rad·s-1 at 0.3 % strain.

The failure of the vGP plot to superpose at a wide range of temperatures suggests that the measurement of degree of chain branching is valid. These systems are becoming increasingly branched with processing and temperatures well above traditional mechanical recycling parameters exacerbate this change. This highlights the importance of using a fixed temperature relevant to PE processing to ensure relevance of the results.
